# Interim Estimates of 2024–2025 Seasonal Influenza Vaccine Effectiveness in Germany—Data From Primary Care and Hospital Sentinel Surveillance

**DOI:** 10.1111/irv.70115

**Published:** 2025-05-06

**Authors:** Annika Erdwiens, Carolin Hackmann, Marianne Wedde, Barbara Biere, Janine Reiche, Ute Preuß, Kristin Tolksdorf, Silke Buda, Ralf Dürrwald

**Affiliations:** ^1^ Department 3 Infectious Disease Epidemiology, Unit 36 Respiratory Infections Robert Koch Institute Berlin Germany; ^2^ Department 1 Infectious Diseases, Unit 17 Influenza Viruses and Other Respiratory Viruses Robert Koch Institute Berlin Germany

**Keywords:** hospitalization, influenza, primary care, vaccine, vaccine effectiveness

## Abstract

Between October 2024 and February 2025, influenza A(H1N1)pdm09 initially predominated in Germany, with subsequent co‐circulation of influenza B/Victoria. We provide interim estimates of 2024/2025 influenza vaccine effectiveness (VE) in Germany across primary care and secondary care. VE against any influenza in primary care was 31% (95% CI: 1–52). Interim VE was high against influenza B; however, interim estimations indicated a much lower VE against influenza A especially in the adult age group below 60 years. In secondary care, VE against any influenza was 69% (95% CI: 21–88). Our findings support promoting influenza vaccination alongside infection‐preventing behavior and prompt antiviral therapy.

## 1 Introduction

In Europe, an intense influenza virus activity was observed in the 2024/2025 season with ongoing influenza circulation by week 8 2025 [1]. The 2024/2025 influenza epidemic in Germany began at the turn of the year with predominant circulation of influenza A(H1N1)pdm09 viruses. Influenza B/Victoria and influenza A(H3N2) co‐circulated, with increasing influenza B activity as of week 8 2025 [2].

In Germany, the Standing Committee on Vaccination (STIKO) recommends an annual vaccination against seasonal influenza for people aged 60 years and over, individuals with chronic underlying diseases of any age and pregnant women.

We report interim vaccine effectiveness (VE) estimates against symptomatic laboratory‐confirmed influenza at primary care and hospital level from week 40 2024 to week 8 2025.

## 2 Methods

A nationwide test‐negative case–control study was conducted based on the results of the virological surveillance of acute respiratory infections (ARI) by the National Reference Center for Influenza Viruses (NRCI) in Germany. In primary care, physicians or pediatricians recruited and systematically took samples from patients consulting with ARI symptoms (at least one of the following symptoms: sore throat, cough, coryza, or fever). In secondary care, patients were selected for recruitment and swabbing who were hospitalized within the last 48 h and treated for any type of severe acute respiratory infection (SARI) symptoms. Written informed consent was obtained from all study participants. Demographic and clinical information were collected via paper‐based questionnaire [3]. Biological samples were tested by real‐time RT‐PCR for influenza virus detection and subtyping at NRCI. A random sample of influenza virus‐positive specimens were sequenced using whole genome sequencing. ARI or SARI patients who tested positive for influenza virus were defined as cases, those who tested negative for any influenza virus were defined as controls.

We used multivariable logistic regression models to estimate the odds ratio (OR) for vaccination, adjusting for sex, age, presence of an underlying disease and onset date. We calculated VE as 1 − OR × 100. We estimated VE overall and, where possible, by age group and influenza (sub)type.

## 3 Results

### 3.1 Primary Care

**FIGURE 1:**
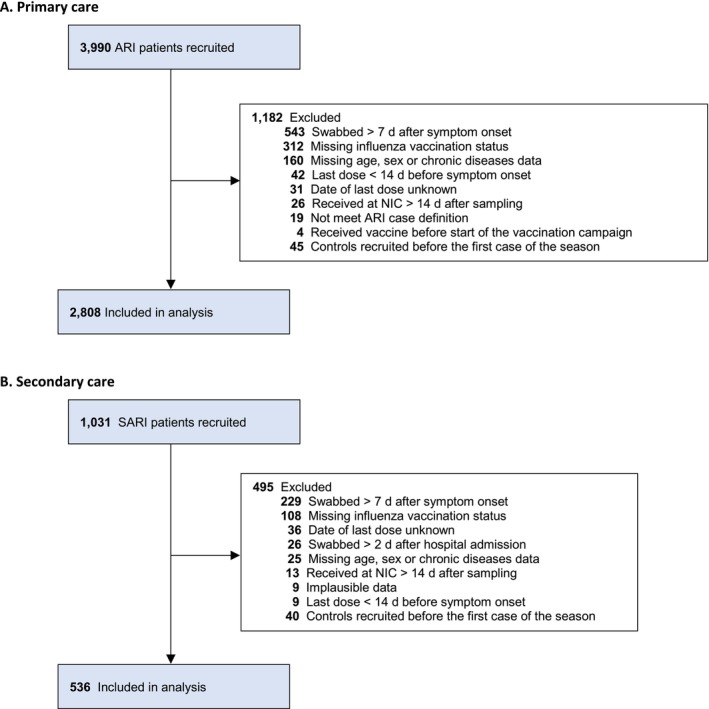
Study restriction flowchart for (A) primary care and (B) secondary care, October 2024–February 2025.

**FIGURE 2:**
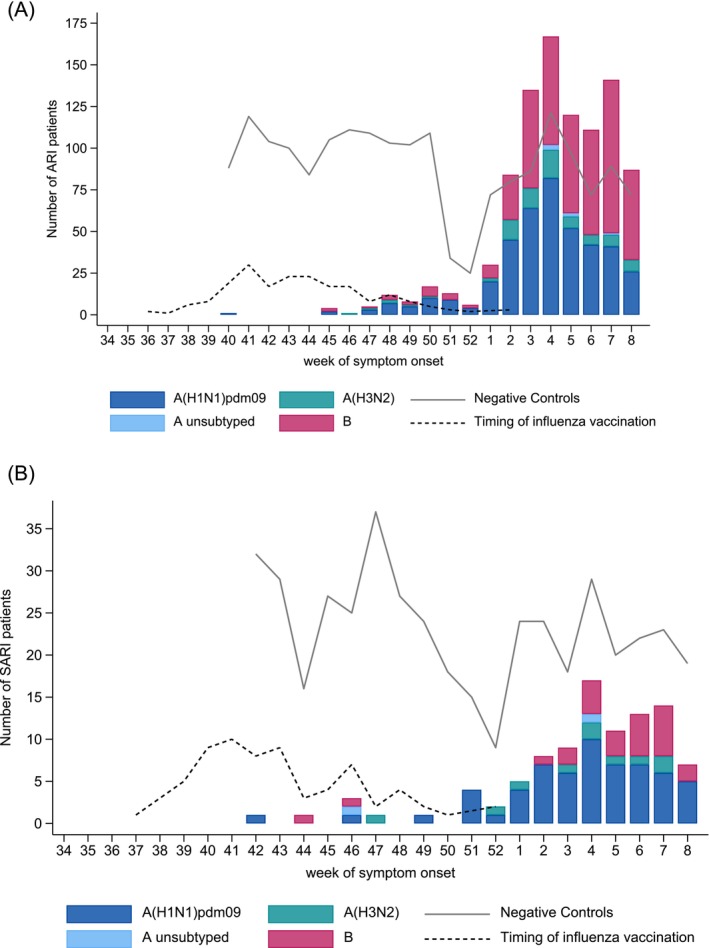
Number of influenza cases and controls by week of symptom onset in (A) primary care and (B) secondary care, October 2024–February 2025. (A) Primary care. (B) Secondary care.

We included 2808 eligible ARI patients for analysis (Figure 1). Overall, there were 926 (33%) laboratory‐confirmed influenza virus infections, of which 413 (45%) were A(H1N1)pdm09 and 76 (8%) A(H3N2). Six (0.7%) influenza A viruses could not be subtyped due to a very low viral load. Four hundred forty‐seven (48%) were influenza B virus infections (Figure 2). There were 15 co‐infections with influenza A(H1N1)pdm09 and influenza B and one co‐infection with influenza A not subtyped and influenza B.

Among the 413 influenza A(H1N1)pdm09 viruses, 35 were sequenced. All were clade 5a.2a viruses, distributed across the following subclades: 28 in C.1.9.3, 6 in C.1.9, and 1 in C.1.9.1. One sequenced influenza A(H3N2) virus was classified as clade 2a.3a.1, subclade J.2. Among the 262 influenza B viruses, 22 were sequenced. All were clade V1A.3a.2 viruses, distributed across following subclades: 13 in C.5.6, 6 in C.5.1, and 3 in C.5.7.

For all ages, VE against any influenza was 31% (95% confidence interval [CI]: 1–52). Among children aged 0–17 years, VE against any influenza was 58% (95% CI: 13–80). In adults aged 18–59 years, VE was 6% (95% CI: −65 to 46) and in those aged 60 years and older 35% (95% CI: −31 to 68). All‐age VE against influenza A was −1% (95% CI: −49 to 31). The all‐age VE against influenza A(H1N1)pdm09 was 2% (95% CI: −48 to 35), −27% (95% CI: −177 to 41) against influenza A(H3N2) and 70% (95% CI: 40–85) against influenza B (Table 1).

**TABLE 1:** Interim adjusted vaccine effectiveness (VE) against all laboratory‐confirmed influenza, influenza A, A(H1N1)pdm09, A(H3N2) and B, by age group, in primary care, October 2024–February 2025.

Influenza (sub)type and setting	Study population	Cases			Controls			IVE^a^	95% CI
		All	Vacc	%	All	Vacc	%		
Primary care									
Influenza A and B	All ages	926	62	7	1882	142	8	31	1 to 52
	0–17 years	543	12	2.2	1047	29	2.8	58	13 to 80
	18–59 years	325	28	9	636	48	8	6	−65 to 46
	≥ 60 years	58	22	38	199	65	33	35	−31 to 68
	< 60 years + chronic diseases	130	16	12	276	33	12	24	−55 to 63
Influenza A	All ages	495	55	11	1882	142	8	−1	−49 to 31
	0–17 years	250	10	4	1047	29	2.8	24	−65 to 65
	18–59 years	193	24	12	636	48	8	−44	−159 to 20
	≥ 60 years	52	21	40	199	65	33	28	−48 to 65
	< 60 years + chronic diseases	79	15	19	276	33	12	−26	−165 to 40
Influenza A(H1N1)pdm09	All ages	413	45	11	1882	142	8	2	−48 to 35
	0–17 years	209	8	3.8	1047	29	2.8	28	−66 to 69
	18–59 years	161	21	13	636	48	8	−49	−175 to 19
	≥ 60 years	43	16	37	199	65	33	33	−43 to 69
	< 60 years + chronic diseases	66	13	20	276	33	12	−32	−188 to 39
Influenza A(H3N2)	All ages	76	10	13	1282	131	10	−27	−177 to 41
Influenza B	All ages	447	10	2.2	1387	131	9	70	40 to 85
Secondary care									
Influenza A and B	All ages	102	7	7	434	63	15	69	21 to 88
	≥ 60 years	30	6	20	180	56	31	76	27 to 92
Influenza A(H1N1)pdm09	All ages	65	7	11	434	63	15	57	−10 to 83
	≥ 60 years	26	6	23	180	56	31	69	4 to 90

^a^ Influenza vaccine effectiveness.

### 3.2 Secondary Care

We recruited 1031 SARI patients. A total of 495 patients were excluded due to missing or implausible data, resulting in 536 eligible SARI patients included for analysis (Figure 1). Overall, there were 102 (19%) laboratory‐confirmed influenza virus infections, of which 65 (64%) were influenza A(H1N1)pdm09, 10 (10%) influenza A(H3N2), and 26 (26%) influenza B virus infections (Figure 2). Two (2%) influenza A isolates could not be subtyped due to a very low viral load. There was one co‐infection with influenza A(H1N1)pdm09 and influenza B.

Three of the 65 influenza A(H1N1)pdm09 viruses were sequenced. All were of clade 5a.2a, subclade C.1.9.3. One of the 26 influenza B viruses was sequenced, belonging to clade V1A.3a.2, subclade C.7.5.

For all ages, VE against any influenza was 69% (95% CI: 21–88), in adults aged 60 years and older 76% (95% CI: 27–92). The all‐age VE against influenza A(H1N1)pdm09 was 57% (95% CI: −10 to 83), in adults aged 60 years and older 69% (95% CI: 4–90) (Table 1).

## 4 Discussion

VE is used to determine the success rate of vaccine use and reflects the proportion of potentially exposed individuals protected from symptomatic influenza after vaccination in comparison to potentially exposed unvaccinated. Factors influencing VE are the composition of vaccine strains, the timing of vaccination in relation to the influenza wave and the strength of the wave, determining the probability of exposure to high viral loads. The higher interim VE against influenza B should therefore be interpreted with caution, as activity of influenza B had not peaked in week 8 2025. Moreover, the B/Victoria vaccine strain is less closely matched to circulating B/Victoria viruses due to the circulation of different subclades of B/Victoria viruses (C.5.1, C.5.6, C.5.7) within clade V1A.3a.2.

In our analyses, interim VE estimates against any influenza among all ages were 31% in primary care. Point estimates were higher in children than in adults, with low VE among individuals aged 18–59 years (6%). In primary care, VE against influenza A was much lower compared to influenza B (−1% vs. 70%), with no detectable VE against influenza A(H1N1)pdm09 (2%). In secondary care, interim VE against any influenza was higher than in primary care (69% vs. 31%). VE was higher among adults 60 years and older than overall (76% for any influenza, 69% for influenza A(H1N1)pdm09).

In the European region, most countries reported co‐circulation of influenza A(H1N1)pdm09, B and A(H3N2) with initial predominance of influenza A(H1N1)pdm09 [4], which is in concordance with our findings from Germany.

The majority of influenza A(H1N1)pdm09 viruses detected in Germany in 2024/25 were of clade 5a.2a and subclade C.1.9 (A/Lisboa/188/2023), thus belonging to a different genetic cluster than the vaccine strain A/Victoria/4897/2022 (clade 5a.2a.1, subclade D). Despite their assignment to different clusters, there is significant cross‐reactivity between the two clades. Studies on the fit of the vaccine strain to circulating influenza viruses using ferret antisera in the hemagglutination inhibition test demonstrated a very good fit: Two third of the isolates reacted within the very good fit range of ±2 log_2_, and only one third of the isolates were just below this range [5].

Our results indicate a lower interim VE against any influenza compared to interim estimates from the 2024/2025 season in the EU (40%–53%) [6]. We report lower VE against influenza A(H1N1)pdm09 compared to Canada (53%) and the EU (30%–72%) [6, 7]. Compared to our end‐of‐season analysis for the 2023/2024 season (VE 39% for influenza A(H1N1)pdm09 in primary care), our interim VE for the 2024/2025 season appears lower [8]. VE against influenza B was comparable to results from the EU (58–74%) and France (75%) [6, 9]. In secondary care, our results indicate slightly higher interim VE than European results both for VE against any influenza (34%–60%) and influenza A(H1N1)pdm09 (28%–53%) [6]. In our end‐of‐season analysis for the 2023/2024 season, VE against any influenza was 65% for all ages and adults 60 years and older, which is also slightly lower than this seasons interim results [8].

The low VE values might be a result of application deficiencies, that is, vaccination too early, since the fit of vaccine strains seemed to be sufficient. Most vaccinations were given between weeks 40 and 47 2024, which was 2–3 months before the peak of the influenza season. There are indications that VE may decline after 2 months, especially in the event of high viral load exposure [10]. To minimize exposure to high viral loads, additional infection control measures should be used whenever possible and close contact with symptomatic individuals should be avoided, regardless of vaccination status [11]. However, VE against severe course of disease appeared to be higher, even with an interval between vaccination and illness onset of 2–3 months.

Limitations include small sample size especially in secondary care and in primary care for certain age groups. Unmeasured confounding, selection bias and recall bias cannot be ruled out. Generally, vaccination coverage was low. The exact vaccination date in secondary care was often unclear due to reliance on self‐reported data, and was therefore imputed as a randomly chosen day (1–30) of the respective reported month. The influenza season in Germany is still ongoing, and end‐of‐season analyses will provide more precise VE estimates against different influenza subtypes and clades or age groups.

## 5 Conclusion

In our analyses, we found low influenza VE against mild laboratory‐confirmed influenza in the German population, with higher VE against severe courses of influenza. Our findings support that influenza vaccination should be promoted alongside other preventive measures, such as encouraging infection‐preventing behavior. Generally, even in vaccinated ARI patients, influenza disease and prompt antiviral therapy should be considered, especially for those at risk of severe disease.

## Author Contributions

**Annika Erdwiens:** conceptualization, data curation, formal analysis, investigation, methodology, project administration, resources, software, validation, visualization, writing – original draft, writing – review and editing. **Carolin Hackmann:** conceptualization, investigation, writing – original draft, writing – review and editing, visualization, validation, methodology, software, formal analysis, project administration, data curation, resources. **Marianne Wedde:** data curation, formal analysis, investigation, methodology, resources, writing – review and editing. **Barbara Biere:** methodology, investigation, writing – review and editing, formal analysis, data curation, resources. **Janine Reiche:** funding acquisition, methodology, investigation, writing – review and editing, formal analysis, data curation, resources. **Ute Preuß:** methodology, investigation, writing – review and editing, formal analysis, data curation, resources. **Kristin Tolksdorf:** conceptualization, methodology, investigation, writing – review and editing, formal analysis, data curation, resources. **Silke Buda:** conceptualization, data curation, formal analysis, funding acquisition, writing – review and editing, investigation, methodology, project administration, supervision, resources. **Ralf Dürrwald:** data curation, formal analysis, funding acquisition, investigation, methodology, resources, writing – review and editing.

## Ethics Statement

The German ARI and SARI Surveillance received ethical approval from the Charité–Universitätsmedizin Berlin Ethical Board (EA2/126/11, EA2/218/19).

## Conflicts of Interest

The authors declare no conflicts of interest.

### Peer Review

The peer review history for this article is available at https://www.webofscience.com/api/gateway/wos/peer‐review/10.1111/irv.70115.

## Data Availability

The data are not publicly available due to privacy or ethical restrictions.
